# Phenotypic Associations of Early Cardiovascular Surgery in Pediatric Marfan Syndrome

**DOI:** 10.3390/children13040504

**Published:** 2026-04-03

**Authors:** Veronika C. Stark, Thomas S. Mir, Caja Langkat, Daniel Biermann, Johanna J. Kanitz, Gerhard Schoen, Yskert von Kodolitsch, Nora Lang, Rainer Kozlik-Feldmann, Michael Huebler, Jakob Olfe

**Affiliations:** 1 Pediatric Cardiology, Clinic for Pediatric Heart Medicine and Adults with Congenital Heart Disease, University Heart and Vascular Center, University Medical Center Hamburg-Eppendorf, 20246 Hamburg, Germanyj.olfe@uke.de (J.O.); 2 Pediatric Cardiac Surgery, Clinic for Pediatric Heart Medicine and Adults with Congenital Heart Disease, University Heart and Vascular Center, University Medical Center Hamburg-Eppendorf, 20246 Hamburg, Germany; 3 Institute of Medical Biometry and Epidemiology, University Medical Center Hamburg-Eppendorf, 20246 Hamburg, Germany; 4 Clinic for Heart Surgery and Vascular Surgery, Herz- und Diabeteszentrum NRW, Ruhr-Universität Bochum, 32545 Bad Oeynhausen, Germany

**Keywords:** Marfan syndrome, aortic root, aortic aneurysm, phenotype, cardiovascular surgical procedures, child, adolescent, risk factors

## Abstract

**Highlights:**

**What are the main findings?**

- This study extends existing knowledge by linking systemic phenotype severity to cardiovascular outcomes.
- Extracardiac features (Marfan-type facial features, pectus carinatum, pes planus, hindfoot deformity, and myopia ≥ 3 diopters) and higher systemic scores are associated with early cardiac surgery in pediatric Marfan syndrome.

**What is the implication of the main finding?**

- This study enables personalized monitoring, preventive care, and timely surgical intervention to improve outcomes in children with Marfan syndrome.

**Abstract:**

**Background/Objectives**: Cardiovascular manifestations in pediatric Marfan syndrome (MFS) exhibit substantial heterogeneity. Early identification of patients at elevated risk of requiring cardiac surgery is essential to optimizing outcomes. This study aimed to determine phenotypic features associated with cardiovascular surgery in genetically confirmed pediatric MFS. **Methods**: Among the 1006 children evaluated, 214 with genetically verified MFS were included in the analysis. We categorized patients by the presence or absence of cardiac surgery during childhood. Systemic and cardiovascular features were assessed. We applied binary logistic regression to identify independent associated manifestations with surgical intervention. **Results**: 20/214 patients (9.3%, 11.5 ± 5.5 years) underwent cardiac surgery (50% aortic root replacement, 20% mitral valve surgery, 30% combined interventions). Extracardiac features—Marfan-type facial features, pectus carinatum, pes planus, hindfoot deformity, and myopia of ≥3 diopters—were significantly associated with an increased probability of surgery (OR 3.0–4.6). Tricuspid valve prolapse and pulmonary artery dilatation were more prevalent in surgical patients. Surgical patients exhibited higher systemic manifestation scores (9.2 vs. 5.2; *p* ≤ 0.05) per revised Ghent criteria (RGC). **Conclusions**: A higher systemic score (RGC) correlates with increased risk for surgery. Marfan-type facial features, pectus carinatum, pes planus, hindfoot deformity, and myopia ≥ 3 diopters were strongly associated with the need for early cardiac surgery. Comprehensive phenotypic assessment, including systemic manifestation scoring, enables risk stratification and supports timely surgical planning in pediatric MFS.

## 1. Introduction

Marfan syndrome (MFS) is the most common hereditary syndromic aortopathy, with an estimated prevalence of 1 in 5000 to 1 in 10,000 individuals [1], although lower reported rates in some regions, particularly in Asia, likely reflect underdiagnosis rather than true differences in prevalence. MFS is caused by pathogenic variants in the *FBN1* gene (chromosome 15q21), leading to structural defects in fibrillin-1 and disruption of the extracellular matrix. In addition, impaired regulation of transforming growth factor–β (TGF-β) signaling contributes to the cardiovascular and systemic manifestations of the disease. Despite its monogenic origin, MFS exhibits marked phenotypic variability, which is influenced by modifier genes, epigenetic mechanisms, and environmental factors, including intrauterine influences [2,3,4,5,6,7].

**Figure 1:**
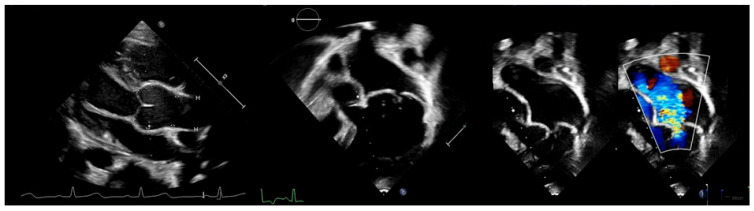
Echocardiographic findings in pediatric Marfan patients. Legend: Aortic root dilatation (**left**), mitral valve prolapse (**middle**), and mitral valve prolapse with regurgitation (**right**); own data, with permission.

Cardiovascular involvement is the leading cause of morbidity and mortality in MFS and often manifests early in life. Aortic root dilatation is the predominant feature, affecting 56% to 71% of pediatric patients, and may progress to life-threatening complications such as aortic dissection (Figure 1 Left) [8,9,10]. Mitral valve prolapse with regurgitation is the second most common cardiac manifestation, with increasing prevalence throughout childhood (17%) and adolescence (75%), and may also require early surgical intervention (Figure 1, middle and right) [11]. Current risk stratification relies primarily on aortic root dimensions and growth velocity; however, these parameters do not adequately capture interindividual variability in disease severity.

Recent studies underscore persistent challenges in elucidating the determinants of phenotypic variability, particularly with respect to cardiovascular risk. Genetic modifiers and environmental influences on developmental pathways and TGF-β signaling are increasingly recognized but remain incompletely understood. These gaps highlight the need for additional, clinically accessible markers to improve early risk assessment.

Systemic manifestations reflect the multisystem connective tissue fragility in MFS and are incorporated into diagnostic frameworks such as the revised Ghent criteria (RGC) [3,12]. However, their prognostic relevance for cardiovascular outcomes in pediatric patients remains unclear, although extracardiac features may reflect underlying disease severity.

The present study aims to characterize pediatric MFS patients undergoing cardiovascular surgery and to identify cardiac and extracardiac manifestations associated with early surgical intervention, thereby improving risk stratification and individualized management in children with MFS.

## 2. Subjects and Methods

### 2.1. Study Design and Population

This retrospective case–control study aimed to identify pediatric patients with MFS at increased risk of requiring cardiovascular surgery during childhood. All subjects were recruited from a specialized center for genetic aortopathies. The study cohort was divided into two groups: patients who underwent cardiovascular surgery during childhood and those who did not. This design was chosen to compare phenotypic characteristics between these groups and to identify the clinical features associated with early surgical intervention.

### 2.2. Inclusion and Exclusion Criteria

Patients aged ≤21 years with a confirmed diagnosis of MFS according to the RGC in combination with a heterozygous pathogenic *FBN1* variant were included [12].

Patients were excluded if they had the following:

(i) Missing genetic confirmation of *FBN1* pathogenicity (*n* = 459);
(ii) Negative or inconclusive genetic testing (*n* = 241);
(iii) Alternative or overlapping connective tissue disorders (*n* = 92).

### 2.3. Data Collection and Study Groups

For patients who underwent cardiovascular surgery, all phenotypic and echocardiographic data were obtained from the last clinical assessment prior to surgery (preoperative evaluation), reflecting the clinical status at the time of surgical decision-making. For patients without surgical intervention, the most recent clinical follow-up visit was used as the reference time point. This approach was chosen to compare patients at clinically meaningful stages rather than at arbitrary chronological time points.

At each visit, standardized assessments included medical history, physical examination, and echocardiography. Data collected comprised demographic characteristics, comorbidities, and systemic manifestations according to the RGC [12]. Patients were regularly evaluated by ophthalmology and orthopedic specialists, with additional consultations as required. Pectus carinatum and other skeletal features were recorded as dichotomous variables (present/absent) according to the revised Ghent criteria and were not graded in severity. Magnetic resonance imaging (MRI) of the vessels and dura mater was performed when feasible without sedation.

### 2.4. Echocardiographic Assessment

Echocardiographic evaluation included an assessment of mitral and tricuspid valve prolapse and regurgitation, aortic valve regurgitation, bicuspid aortic valve, and measurements of the aortic annulus, sinus of Valsalva, sinotubular junction, ascending aorta, and pulmonary artery. Measurements were obtained in parasternal long- and short-axis views using the inner edge-to-inner edge technique at systole. Z-scores were calculated according to Pettersen et al. [13], with ascending aorta Z-scores additionally assessed using Gautier et al. [14]. All examinations were performed by the same experienced team specializing in genetic aortopathies.

### 2.5. Statistical Analysis

Continuous variables are presented as mean ± standard deviation (SD), and categorical variables as counts and percentages. Group comparisons were performed using unpaired *t*-tests and Fisher’s exact tests, as appropriate. Binary logistic regression analysis was conducted to identify manifestations associated with early cardiovascular surgery.

The systemic score according to the RGC was first analyzed as a continuous variable. Subsequently, individual systemic features were evaluated. A backward stepwise selection procedure was applied to derive a parsimonious model including only variables significantly associated with surgical intervention. The following variables were considered: typical facial features, wrist/thumb sign, pectus excavatum, pectus carinatum, scoliosis, pes planus, hindfoot deformity, ectopia lentis, myopia, pneumothorax, and striae atrophicae. These variables were selected to cover the major organ systems defined by the RGC while limiting the number of parameters to reduce the risk of statistical overfitting. Dural ectasia was excluded due to incomplete MRI data in younger patients.

A *p*-value < 0.05 was considered statistically significant. Data were stored using FileMaker Pro, and statistical analyses were performed with Microsoft Excel 365 (Microsoft Corporation, Microsoft Excel 365 for Enterprise, Version 2304 [Computer software]; Redmond, WA, USA: Microsoft Corporation; 2024) and SPSS (IBM Corp., IBM SPSS Statistics for Windows, Version 29.0.2.0 [Computer software]; Armonk, NY, USA: IBM Corp.; 2023). During the preparation of this manuscript, the authors used ChatGPT (OpenAI, Version 5.3; San Francisco, CA, USA; 2026) for the purposes of illustrating the features.

### 2.6. Ethical Considerations

The study was registered, designed, performed, and controlled according to current guidelines of Good Clinical Practice and approved by the local ethics committee (Ethics Committee Hamburg (PV 5457)). Informed consent was obtained from all participants or their legal guardians.

## 3. Results

### 3.1. Cohort Characteristics

A total of 1006 children evaluated for genetic aortopathies were screened. Of these, 214 patients with genetically confirmed MFS were included. Among them, 20 patients (9.3%) underwent cardiovascular surgery during childhood.

Within the surgical group, 10 patients (50%; mean age 14.0 ± 5.5 years) underwent isolated valve-sparing aortic root replacement (David procedure), 4 patients (20%; 11.7 ± 0.8 years) underwent isolated mitral valve surgery, and 5 patients (25%; 10.7 ± 5.0 years) underwent combined aortic root and mitral valve surgery. One patient underwent mitral valve surgery following prior aortic root replacement, at ages 5.9 and 13.8 years, respectively (5%). The overall mean age at surgery was 11.5 ± 5.5 years (range 0.3–21.1), and 70% of operated patients were male.

The remaining 194 patients did not undergo surgical intervention (mean age 12.5 ± 5.7 years; 47.4% male). No significant differences in age or sex distribution were observed between the groups. The mean follow-up duration in the non-surgical group was 12.7 ± 5.7 years. No significant differences in age or sex distribution were observed between the surgical and non-surgical groups.

### 3.2. Echocardiographic Findings in Surgical Patients

Echocardiographic characteristics differed between surgical subgroups (Table 1 and Table 2). Patients undergoing aortic root surgery exhibited significantly higher z-scores of the sinus of Valsalva compared to those undergoing isolated mitral valve surgery (4.8 ± 1.4 vs. 3.0 ± 0.7; *p* = 0.015). Conversely, patients undergoing mitral valve surgery showed higher z-scores of the mitral valve annulus compared to patients undergoing David procedures; however, this difference did not reach statistical significance (3.7 ± 1.8 vs. −0.2 ± 1.8; *p* = 0.15), likely due to the very small sample size. Left ventricular fractional shortening was numerically lower in the mitral valve surgery group compared to the David group (30.3% vs. 36.1%), but this difference did not reach statistical significance (*p* = 0.40) and should be interpreted with caution due to limited sample size.

### 3.3. Phenotypic Differences Between Patients with and Without Surgery

**Figure 2:**
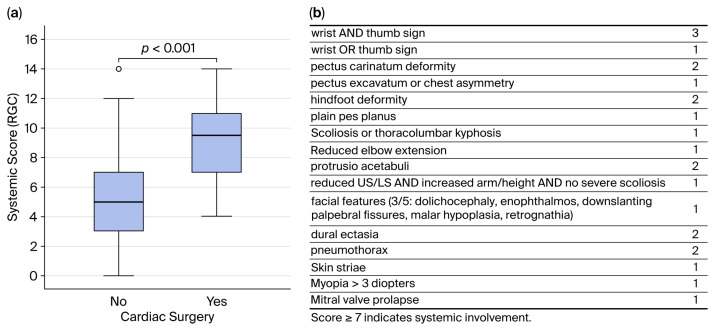
Systemic manifestations (according to the revised Ghent criteria (RGC)) in pediatric Marfan patients: (**a**) Box plot of the systemic manifestation score in both study cohorts. (**b**) Score for systemic manifestations according to the RGC. A score of ≥7 indicates systemic manifestations. The maximum possible score is 20 points [12]; US/LS, upper segment/lower segment.

Patients who underwent cardiovascular surgery had a significantly higher prevalence of systemic involvement (85% vs. 36%; *p* ≤ 0.05) and a significantly higher systemic manifestation score according to the revised Ghent criteria (9.2 ± 2.6 vs. 5.2 ± 3.1; *p* ≤ 0.05) (Figure 2).

As shown in Table 3, several extracardiac features were significantly more prevalent in operated patients, including Marfan-typical facial dysmorphism, enophthalmos, the wrist sign, reduced elbow extension, pectus carinatum, pes planus, hindfoot deformity, myopia ≥ 3 diopters, a high-arched palate, and dural ectasia. In addition, cardiovascular manifestations such as aortic aneurysm, mitral valve prolapse, mitral regurgitation, tricuspid valve prolapse, aortic regurgitation, and pulmonary artery dilatation were more frequent in the surgical group. Patients requiring surgery also exhibited earlier manifestation of selected features, including wrist sign, pectus carinatum, increased arm-span-to-height ratio, and striae atrophicae.

### 3.4. Association Between Systemic Phenotype and Cardiovascular Involvement

Analysis of the systemic manifestation score as a continuous variable demonstrated a dose–response relationship with early cardiovascular surgery. Each additional point in the systemic score was associated with a 1.54-fold increase in the odds of surgical intervention (95% CI: 1.27–1.85; *p* < 0.05).

Higher systemic manifestation scores were also associated with increased cardiovascular involvement. In particular, higher scores were associated with larger aortic root dimensions, reflected by increased Z-scores of the sinus of Valsalva (*p* ≤ 0.05). Patients with valve regurgitation tended to have higher systemic scores, although these associations did not consistently reach statistical significance. No clear association was observed between systemic scores and left ventricular dimensions.

### 3.5. Multivariable Analysis of Phenotypic Features

**Figure 3:**
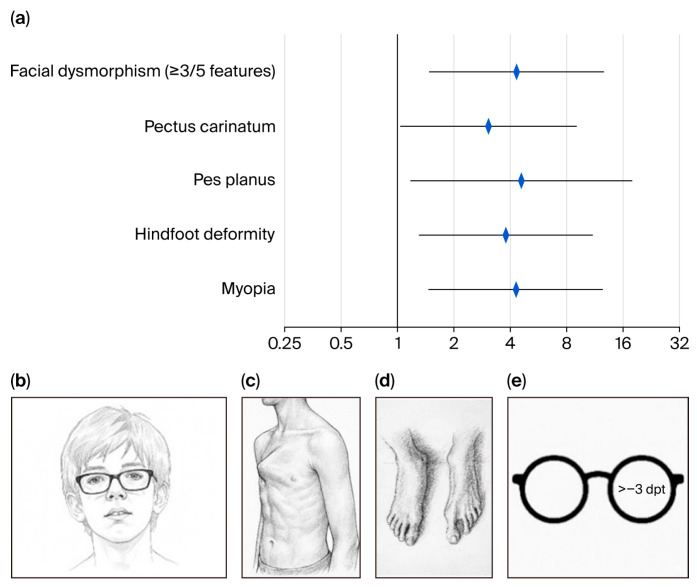
Phenotypic features in pediatric Marfan patients undergoing cardiovascular surgery: (**a**) Forest plot of logistic regression results after backward selection (OR, 95% CI). (**b**–**e**) Marfan manifestations that are associated with cardiac surgery in childhood: (**b**) facial dysmorphism, (**c**) pectus carinatum, (**d**) pes planus/hindfoot deformity, and (**e**) myopia.

A backward stepwise logistic regression analysis identified five extracardiac features independently associated with early cardiovascular surgery: facial dysmorphism (OR 4.33; 95% CI: 1.48–12.70; *p* = 0.01), pectus carinatum (OR 3.07; 95% CI: 1.04–9.07; *p* = 0.04), pes planus (OR 4.57; 95% CI: 1.17–17.83; *p* = 0.03), hindfoot deformity (OR 3.78; 95% CI: 1.30–10.97; *p* = 0.02), and myopia of ≥3 diopters (OR 4.27; 95% CI: 1.46–12.45; *p* = 0.01) (Figure 3). Among these, the strongest associations were observed for pes planus, facial dysmorphism, and myopia.

### 3.6. Genetic Findings in Operated Patients

Genetic data of operated patients are summarized in Table 4. Among the patients with available detailed genetic findings, the majority (65%) carried *FBN1* missense variants, of which 69% involved cysteine substitutions. No formal association analysis between genotype and surgical outcome was performed due to the limited sample size.

## 4. Discussion

This study shows that a higher systemic phenotypic burden and specific extracardiac features are associated with early cardiovascular surgery in pediatric MFS. Cardiovascular complications remain the principal determinant of morbidity and mortality in pediatric MFS. Although aortic root dimensions and growth rates are established parameters for risk assessment, clinically accessible markers associated with early surgical intervention during childhood are limited.

In our cohort of genetically confirmed pediatric MFS patients, 9.3% required cardiovascular surgery during childhood, consistent with previously reported rates ranging from 6% to 26% [11,15]. In line with earlier observations [16], no cases of aortic dissection occurred, underscoring the benefit of structured surveillance and timely elective intervention in specialized centers. Genotype–phenotype correlations have identified specific *FBN1* variants, particularly cysteine-altering missense mutations, as markers of more severe cardiovascular disease [17,18,19,20]. The predominance of such variants in our surgical cohort is consistent with these findings. However, as genetic testing may not directly guide clinical decision-making, our analysis focused on clinically accessible phenotypic markers.

A central finding of this study is the strong association between systemic phenotypic burden and early surgical intervention. The systemic score (RGC), analyzed as a continuous variable, demonstrated a dose–response relationship, with increasing systemic burden associated with a higher likelihood of surgery. These findings highlight the importance of considering systemic involvement quantitatively rather than dichotomously.

Importantly, these associations should be interpreted in the context of phenotypic diversity in MFS. As highlighted by Xu et al. [3], considerable interindividual variability exists in the clinical expression of MFS, driven by genetic, epigenetic, and environmental modifiers. In this context, higher systemic scores and the identified extracardiac features likely reflect a subgroup of patients with a more severe or more systemically expressed phenotype, rather than acting as independent predictors of surgical outcomes. This interpretation is supported by our additional analyses demonstrating that higher systemic manifestation scores are associated with increased cardiovascular involvement, particularly larger aortic root dimensions.

Consistent with this concept, five extracardiac features—Marfan-type facial dysmorphism, pectus carinatum, pes planus, hindfoot deformity, and myopia of ≥3 diopters—were independently associated with early surgery. While thoracic deformities have been linked to aortic root dilatation [21,22] and striae atrophicae to a potential risk of dissection [23], the predictive value of extracardiac manifestations for surgical endpoints remains controversial. Notably, the Pediatric Heart Network Marfan Trial did not identify extracardiac features as predictors of a severe cardiac phenotype [24]. Differences in study design and endpoints likely explain this discrepancy, as prior studies used broader definitions of disease severity and alternative scoring systems based on the original Ghent nosology, whereas our study applied the RGC.

In addition to extracardiac markers, we observed a higher prevalence of tricuspid valve prolapse and pulmonary artery dilatation in operated patients, supporting the concept of a more extensive cardiovascular phenotype [25,26]. Current recommendations emphasize aortic root growth velocity, aortic valve dysfunction, family history of aortic surgery or dissection in childhood, pregnancy, arterial tortuosity, and early-onset MFS as primary determinants for surgical timing [27]. Additional parameters include older age, taller stature, elevated systolic blood pressure, larger baseline aortic root diameter, and generalized proximal aortic enlargement [28]. Also, morphologic abnormalities of the proximal aorta—particularly involving the sinotubular junction and ascending aorta—are risk factors for higher cardiovascular involvement, as demonstrated in a large pediatric cohort [29]. However, these parameters alone do not fully capture disease heterogeneity. While emerging approaches—including genotype–phenotype correlations, circulating biomarkers, and advanced imaging—may refine future risk stratification [30], many are not yet routinely applicable in pediatric practice.

Our findings suggest that readily assessable systemic phenotypic features may provide an additional, pragmatic layer of clinical information that complements established cardiovascular risk markers. Given the low perioperative risk and favorable long-term outcomes of pediatric cardiac surgery in MFS, early risk-adapted management remains essential to prevent acute complications such as aortic dissection [31,32,33].

This study has limitations. The relatively small number of surgical events limits statistical power and may affect the robustness of multivariable analyses, although this reflects the low incidence of early surgery in pediatric MFS. The retrospective, single-center design may limit generalizability. In addition, the identified associations should not be interpreted as causal or predictive but rather as reflecting underlying disease severity. Not all dimensions of phenotypic diversity described in recent studies, including genetic, epigenetic, and environmental modifiers, were systematically assessed [3]. Thus, our findings reflect selected clinically accessible features and may not fully capture the complexity of phenotypic variability in MFS. We deliberately included all pediatric patients who underwent surgery and did not analyze early-onset MFS separately, as the limited number of events precluded meaningful subgroup analyses. Instead, we focused on identifying phenotypic characteristics associated with an increased risk of surgery during childhood across the broader pediatric MFS population. A key strength of this study is the homogeneous cohort of genetically confirmed patients who underwent standardized clinical and echocardiographic assessment in a specialized aortopathy clinic. Future prospective studies are warranted to determine whether these associations translate into true predictive markers of early cardiovascular intervention and to explore additional systemic features beyond the RGC for their potential predictive value [34].

## 5. Conclusions

In pediatric MFS, a higher systemic phenotypic burden and selected extracardiac features are associated with early cardiovascular surgery. A higher systemic score (RGC) shows a clear dose–response association with surgical intervention, and five easily assessable extracardiac features are independently associated with early surgery (Marfan-type facial features, pectus carinatum, pes planus, hindfoot deformity, and myopia of ≥3 diopters). These findings suggest that systemic phenotypic features reflect a more advanced disease phenotype rather than acting as independent predictors of future surgery. Comprehensive phenotypic assessment may therefore help identify patients who could benefit from closer echocardiographic surveillance and timely surgical evaluation.

## Figures and Tables

**Table 1:** Echocardiographic findings in pediatric Marfan patients with isolated David surgery (*n* = 10).

	**Mean**	**Standard Deviation**	**Z-Score ± SD**	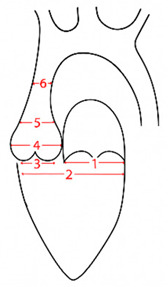
1 Mitral valve annulus ^1^	35.5	5.8	−0.2 ± 1.8	
2 LVIDd ^1^	54.9	4.2	0.9 ± 1.5	
3 Aortic valve annulus ^1^	27.2	3.6	3.0 ± 2.8	
4 Sinuses of Valsalva ^1^	43.5	5.0	4.8 ± 1.4	
5 Sinotubular junction ^1^	32.0	4.6	2.9 ± 1.9	
6 Aorta ascendens ^2^	27.0	5.0	1.7 ± 1.5	
FS (%)	36.1	4.1	-	
Aortic regurgitation (≥2)	-	-	-	2/10, 20%

Mean, value (mm); SD, standard deviation (mm); LVIDd, left ventricular dimension in end diastole; FS, fractional shortening. ^1^ Z-score calculated according to Pettersen et al. [13]. ^2^ Z-score calculated according to Gautier et al. [14].

**Table 2:** Echocardiographic findings in pediatric Marfan patients with isolated mitral valve surgery (*n* = 4).

	**Mean**	**Standard Deviation**	**Z-Score ± SD**	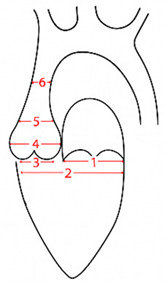
1 Mitral valve annulus ^1^	33.5	6.4	3.7 ± 1.8	
2 LVIDd ^1^	48.5	16.3	4.3 ± 1.4	
3 Aortic valve annulus ^1^	19.8	5.9	2.9 ± 0.4	
4 Sinuses of Valsalva ^1^	27.0	7.3	3.0 ± 0.7	
5 Sinotubular junction ^1^	19.5	5.6	2.0 ± 0.5	
6 Aorta ascendens ^2^	17.0	5.1	0.2 ± 0.9	
FS (%)	30.2	11.7	-	
Aortic regurgitation (≥2)	-	-	-	0/4, 0%

Mean, value (mm); SD, standard deviation (mm); LVIDd, left ventricular dimension in end diastole; FS, fractional shortening. ^1^ Z-score calculated according to Pettersen et al. [13]. ^2^ Z-score calculated according to Gautier et al. [14].

**Table 3:** Prevalence and manifestation age of symptoms in patients with/without cardiac surgery in pediatric Marfan patients (*n* = 214).

	Without Cardiac Surgery (*n* = 194)		With Cardiac Surgery (*n* = 20)	
	Prevalence (%)	Age (Years)	Prevalence (%)	Age (Years)
Systemic manifestation ^a,^*	36	12.2 ± 4.4	85	10.2 ± 5.6
Facial dysmorphism ^b,^*	29	7.3 ± 5.3	60	6.9 ± 6.4
Dolichocephaly	41	8.4 ± 5.7	45	6.1 ± 6.5
Malar hypoplasia	24	8.1 ± 5.0	35	11.3 ± 4.8
Downslanting palpebral fissure	45	8.1 ± 5.9	60	6.6 ± 4.9
Retrognathia	30	7.3 ± 5.9	45	5.1 ± 6.4
Enophthalmos *	24	8.0 ± 5.7	50	7.5 ± 6.4
Thumb sign	55	10.4 ± 5.0	75	7.8 ± 4.7
Wrist sign *^,†^	50	11.7 ± 4.6	75	9.0 ± 3.7
Wrist + thumb sign	45	11.5 ± 4.7	65	8.8 ± 3.8
Reduced elbow extension *	7	11.6 ± 4.8	25	9.0 ± 7.6
Pectus excavatum	20	9.7 ± 5.6	25	7.8 ± 4.8
Chest asymmetry	32	10.0 ± 4.9	40	6.6 ± 4.6
Pectus carinatum *^,†^	19	11.4 ± 4.2	40	6.4 ± 5.1
Scoliosis	38	11.0 ± 4.7	50	10.8 ± 2.7
Protrusio acetabuli	3	9.6 ± 5.5	10	7.2 ± 0.4
Arm-span/height ratio ≥ 1.05 ^†^	27	11.4 ± 4.6	40	6.4 ± 4.8
Pes planus *	53	9.3 ± 4.9	85	8.1 ± 5.0
Hindfoot deformity *	30	8.3 ± 4.9	65	9.3 ± 5.7
Ectopia lentis	23	8.5 ± 5.4	45	8.0 ± 6.1
Myopia *	20	8.4 ± 5.4	50	7.4 ± 3.2
Pneumothorax	4	15.4 ± 0.8	5	no data
Striae atrophicae ^†^	28	14.4 ± 2.6	50	11.4 ± 4.9
High arched palate *	45	10.8 ± 4.7	80	8.7 ± 5.4
Aortic aneurysm *	47	8.7 ± 5.4	100	7.5 ± 5.7
Pos. family history	57	n/a	35	n/a
Mitral valve prolapse *	73	8.8 ± 5.4	100	7.7 ± 5.7
Mitral valve regurgitation *	64	8.8 ± 5.8	100	8.5 ± 6.1
Tricuspid valve prolapse *	83	8.2 ± 5.5	100	8.1 ± 5.7
Tricuspid valve regurgitation	93	8.4 ± 5.8	100	8.1 ± 5.7
Aortic valve regurgitation *	15	10.6 ± 5.6	90	9.9 ± 5.6
Bicuspid aortic valve	9	n/a	5	n/a
Pulmonary artery dilatation *	12	11.4 ± 4.4	35	13.2 ± 3.5
Dural ectasia ^c,^*	56	12.0 ± 3.9	92	13.0 ± 4.6

n/a, not applicable. ^a^ Systemic manifestation: patients with a score ≥7 regarding the revised Ghent criteria (RGC). ^b^ Facial dysmorphism: ≥3/5 features regarding RGC. ^c^ Dural ectasia: without cardiac surgery (*n* = 94) and with cardiac surgery (*n* = 12). * *p* ≤ 0.05 concerning prevalence; ^†^ *p* ≤ 0.05 concerning age at manifestation.

**Table 4:** *FBN1* variants in operated Marfan patients (n = 20). The asterisk (*) denotes a stop codon, and the number “19” indicates that a premature stop codon occurs 19 amino acids downstream of the first altered amino acid (valine) at position 2347.

Nr.	Gen	Exon/Intron	Mutation	cDNA	Protein
1	*FBN1*	Exon 12	Nonsense	c.1546C > T	p.Arg516Ter
2	*FBN1*	Exon 28	In-frame deletion	c.3338-3_3346del	no data
3	*FBN1*	Exon 33	Missense	c.4172G > T	p.Cys1391Phe
4	*FBN1*	no data	no data	no data	no data
5	*FBN1*	Exon 52	Missense	c.6388G > A	p.Glu2130Lys
6	*FBN1*	Exon 24	Missense	c.2849G > T	p.Cys950Phe
7	*FBN1*	Exon 29	Missense	c.3706T > C	p.Cys1236Arg
8	*FBN1*	Exon 47–49	no data	no data	no data
9	*FBN1*	Exon 61	Missense	c.7648T > G	p.Cys2550Gly
10	*FBN1*	Exon 31	Missense	c.4022A > T	p.Asn1341Ile
11	*FBN1*	Exon 29	Missense	c.3598G > A	p.Glu1200Lys
12	*FBN1*	no data	no data	no data	no data
13	*FBN1*	Exon 27	Missense	c.3419G > A	p.Cys1140Tyr
14	*FBN1*	Exon 44	Missense	c.5699G > A	p.Cys1900Tyr
15	*FBN1*	Exon 25	Missense	c.3037G > A	p.Gly1013Arg
16	*FBN1*	Intron 40	Splice-site-mutation	IVS40-1G > A	no data
17	*FBN1*	Exon 31	Missense	c.3850T > C	p.Cys1284Arg
18	*FBN1*	Exon 45	Missense	c.5929T > A	p.Cys1977Ser
19	*FBN1*	Exon 58	Missense	c.7094G > A	p.Cys2365Tyr
20	*FBN1*	Exon 58	Frameshift	c.7039_7040delAT	p.Met2347Valfs*19

## Data Availability

The raw data supporting the conclusions of this article will be made available by the authors upon request. The data are not publicly available due to the inclusion of sensitive patient-level data, where a residual risk of re-identification cannot be fully excluded. The data are available from the corresponding author on reasonable request to ensure appropriate use and interpretation.

## Abbreviations

The following abbreviations are used in this manuscript:

BAV	Bicuspid aortic valve
cDNA	Complementary deoxyribonucleic acid
CI	Confidence interval
*FBN1*	Fibrillin-1 gene
LVIDd	Left ventricular internal diameter in end diastole
MFS	Marfan syndrome
MRI	Magnetic resonance imaging
n/a	Not applicable
OR	Odds ratio
RGC	Revised Ghent criteria
SD	Standard deviation
US/LS	Upper segment/lower segment

## References

[1] Groth K.A., Hove H., Kyhl K., Folkestad L., Gaustadnes M., Vejlstrup N., Stochholm K., Østergaard J.R., Andersen N.H., Gravholt C.H. (2015). Prevalence, Incidence, and Age at Diagnosis in Marfan Syndrome. Orphanet J. Rare Dis..

[2] Dietz H.C., Cutting C.R., Pyeritz R.E., Maslen C.L., Sakai L.Y., Corson G.M., Puffenberger E.G., Hamosh A., Nanthakumar E.J., Curristin S.M. (1991). Marfan Syndrome Caused by a Recurrent de Novo Missense Mutation in the Fibrillin Gene. Nature.

[3] Xu Z., Li Z., Xiang L., Chen T., Lu W., Guo C., Chen J., Kang B., Liu Z., Xiang D. (2025). Phenotypic Diversity of Marfan Syndrome. JACC Adv..

[4] van Andel M.M., Groenink M., van den Berg M.P., Timmermans J., Scholte A.J.H.A., Mulder B.J.M., Zwinderman A.H., de Waard V. (2021). Genome-Wide Methylation Patterns in Marfan Syndrome. Clin. Epigenetics.

[5] Zhang W.Z., Wu C.Y., Lai H. (2025). A Review on the Role of DNA Methylation in Aortic Disease Associated With Marfan Syndrome. Cardiol. Res..

[6] Mehrkens D., Dohr J., Nettersheim F.S., Ballmann F., Bastigkeit J., Brückner A., Geissen S., De Vore L., Fitsch C., Diekmann C. (2026). Myeloperoxidase Aggravates Thoracic Aortic Aneurysm Formation in Marfan Disease. Cardiovasc. Res..

[7] Aubart M., Gazal S., Arnaud P., Benarroch L., Gross M.-S., Buratti J., Boland A., Meyer V., Zouali H., Hanna N. (2018). Association of Modifiers and Other Genetic Factors Explain Marfan Syndrome Clinical Variability. Eur. J. Hum. Genet..

[8] Faivre L., Masurel-Paulet A., Collod-Beroud G., Callewaert B.L., Child A.H., Stheneur C., Binquet C., Gautier E., Chevallier B., Huet F. (2009). Clinical and Molecular Study of 320 Children With Marfan Syndrome and Related Type I Fibrillinopathies in a Series of 1009 Probands With Pathogenic FBN1 Mutations. Pediatrics.

[9] Mueller G.C., Stark V., Steiner K., von Kodolitsch Y., Rybczynski M., Weil J., Mir T.S. (2013). Impact of Age and Gender on Cardiac Pathology in Children and Adolescents With Marfan Syndrome. Pediatr. Cardiol..

[10] Ekhomu O., Naheed Z.J. (2015). Aortic Involvement in Pediatric Marfan Syndrome: A Review. Pediatr. Cardiol..

[11] van Karnebeek C.D.M. (2001). Natural History of Cardiovascular Manifestations in Marfan Syndrome. Arch. Dis. Child..

[12] Loeys B.L., Dietz H.C., Braverman A.C., Callewaert B.L., De Backer J., Devereux R.B., Hilhorst-Hofstee Y., Jondeau G., Faivre L., Milewicz D.M. (2010). The Revised Ghent Nosology for the Marfan Syndrome. J. Med. Genet..

[13] Pettersen M.D., Du W., Skeens M.E., Humes R.A. (2008). Regression Equations for Calculation of Z Scores of Cardiac Structures in a Large Cohort of Healthy Infants, Children, and Adolescents: An Echocardiographic Study. J. Am. Soc. Echocardiogr..

[14] Gautier M., Detaint D., Fermanian C., Aegerter P., Delorme G., Arnoult F., Milleron O., Raoux F., Stheneur C., Boileau C. (2010). Nomograms for Aortic Root Diameters in Children Using Two-Dimensional Echocardiography. Am. J. Cardiol..

[15] Wozniak-Mielczarek L., Sabiniewicz R., Drezek-Nojowicz M., Nowak R., Gilis-Malinowska N., Mielczarek M., Łabuc A., Waldoch A., Wierzba J. (2019). Differences in Cardiovascular Manifestation of Marfan Syndrome Between Children and Adults. Pediatr. Cardiol..

[16] Hascoet S., Edouard T., Plaisancie J., Arnoult F., Milleron O., Stheneur C., Chevallier B., Zordan C., Odent S., Bal L. (2020). Incidence of Cardiovascular Events and Risk Markers in a Prospective Study of Children Diagnosed with Marfan Syndrome. Arch. Cardiovasc. Dis..

[17] Takeda N., Inuzuka R., Maemura S., Morita H., Nawata K., Fujita D., Taniguchi Y., Yamauchi H., Yagi H., Kato M. (2018). Impact of Pathogenic *FBN1* Variant Types on the Progression of Aortic Disease in Patients With Marfan Syndrome. Circ. Genom. Precis. Med..

[18] Schrijver I., Liu W., Brenn T., Furthmayr H., Francke U. (1999). Cysteine Substitutions in Epidermal Growth Factor–Like Domains of Fibrillin-1: Distinct Effects on Biochemical and Clinical Phenotypes. Am. J. Hum. Genet..

[19] Faivre L., Collod-Beroud G., Loeys B.L., Child A., Binquet C., Gautier E., Callewaert B., Arbustini E., Mayer K., Arslan-Kirchner M. (2007). Effect of Mutation Type and Location on Clinical Outcome in 1,013 Probands with Marfan Syndrome or Related Phenotypes and FBN1 Mutations: An International Study. Am. J. Hum. Genet..

[20] Arnaud P., Milleron O., Hanna N., Ropers J., Ould Ouali N., Affoune A., Langeois M., Eliahou L., Arnoult F., Renard P. (2021). Clinical Relevance of Genotype–Phenotype Correlations beyond Vascular Events in a Cohort Study of 1500 Marfan Syndrome Patients with FBN1 Pathogenic Variants. Genet. Med..

[21] De Maio F., Pisano C., Caterini A., Bertoldo F., Ruvolo G., Farsetti P. (2021). Marfan Syndrome in Children: Correlation between Musculoskeletal Features and Cardiac Z-Score. J. Pediatr. Orthop. B.

[22] Rhee D., Solowiejczyk D., Altmann K., Prakash A., Gersony W.M., Stolar C., Kleinman C., Anyane-Yeboa K., Chung W.K., Hsu D. (2008). Incidence of Aortic Root Dilatation in Pectus Excavatum and Its Association With Marfan Syndrome. Arch. Pediatr. Adolesc. Med..

[23] Ágg B., Benke K., Szilveszter B., Pólos M., Daróczi L., Odler B., Nagy Z.B., Tarr F., Merkely B., Szabolcs Z. (2014). Possible Extracardiac Predictors of Aortic Dissection in Marfan Syndrome. BMC Cardiovasc. Disord..

[24] John S., Young L.T., Lacro R.V., Hoskoppal A., Ou Z., Presson A.P., Johnson J.T., Andrade L., Minich L.L., Menon S. (2025). Extracardiac Manifestations Fail to Predict the Severity of Cardiac Phenotype in Children and Young Adults with Marfan Syndrome. Pediatr. Cardiol..

[25] Stark V.C., Huemmer M., Olfe J., Mueller G.C., Kozlik-Feldmann R., Mir T.S. (2018). The Pulmonary Artery in Pediatric Patients with Marfan Syndrome: An Underestimated Aspect of the Disease. Pediatr. Cardiol..

[26] Stark V.C., Olfe J., Pesch J., Tahir E., Weinrich J.M., Wiegand P., Kozlik-Feldmann R., Von Kodolitsch Y., Mir T.S. (2022). Tricuspid Valve Prolapse as an Early Predictor for Severe Phenotype in Children with Marfan Syndrome. Acta Paediatr..

[27] Morris S.A., Flyer J.N., Yetman A.T., Quezada E., Cappella E.S., Dietz H.C., Milewicz D.M., Ouzounian M., Rigelsky C.M., Tierney S. (2024). Cardiovascular Management of Aortopathy in Children: A Scientific Statement From the American Heart Association. Circulation.

[28] Lazarevic A.M., Nakatani S., Okita Y., Marinkovic J., Takeda Y., Hirooka K., Matsuo H., Kitamura S., Yamagishi M., Miyatake K. (2006). Determinants of Rapid Progression of Aortic Root Dilatation and Complications in Marfan Syndrome. Int. J. Cardiol..

[29] Hoskoppal A., Menon S., Trachtenberg F., Burns K.M., De Backer J., Gelb B.D., Gleason M., James J., Lai W.W., Liou A. (2018). Predictors of Rapid Aortic Root Dilation and Referral for Aortic Surgery in Marfan Syndrome. Pediatr. Cardiol..

[30] Stengl R., Ágg B., Pólos M., Mátyás G., Szabó G., Merkely B., Radovits T., Szabolcs Z., Benke K. (2021). Potential Predictors of Severe Cardiovascular Involvement in Marfan Syndrome: The Emphasized Role of Genotype–Phenotype Correlations in Improving Risk Stratification—A Literature Review. Orphanet J. Rare Dis..

[31] Braverman A.C. (2004). Timing of Aortic Surgery in the Marfan Syndrome. Curr. Opin. Cardiol..

[32] Park I., Yang J.-H., Sung K., Jun T.-G., Kang I.-S., Huh J., Song J.Y., Park P.W. (2024). Valve-Sparing Root Replacement in Children with Connective Tissue Disease: Long-Term Risk of Aortic Events. J. Thorac. Cardiovasc. Surg..

[33] Pitcher A., Spata E., Emberson J., Davies K., Halls H., Holland L., Wilson K., Reith C., Child A.H., Clayton T. (2022). Angiotensin Receptor Blockers and β Blockers in Marfan Syndrome: An Individual Patient Data Meta-Analysis of Randomised Trials. Lancet.

[34] von Kodolitsch Y., Demolder A., Girdauskas E., Kaemmerer H., Kornhuber K., Muino Mosquera L., Morris S., Neptune E., Pyeritz R., Rand-Hendriksen S. (2019). Features of Marfan Syndrome Not Listed in the Ghent Nosology–the Dark Side of the Disease. Expert Rev. Cardiovasc. Ther..

